# Individual heterogeneity screened umbilical cord-derived mesenchymal stromal cells with high Treg promotion demonstrate improved recovery of mouse liver fibrosis

**DOI:** 10.1186/s13287-021-02430-6

**Published:** 2021-06-22

**Authors:** Yuanyuan Xie, Shuo Liu, Liudi Wang, Hui Yang, Chenxu Tai, Li Ling, Libo Chen, Shanshan Liu, Bin Wang

**Affiliations:** 1 Clinical Stem Cell Center, The Affiliated Drum Tower Hospital of Nanjing University Medical School, 321 Zhongshan Road, Nanjing, 210000 People’s Republic of China; 2 Department of Endocrinology, University Health Science Center, Hua Zhong University of Science and Technology Union Shenzhen Hospital and The 6th Affiliated Hospital of Shenzhen, Shenzhen, 518052 Guangdong People’s Republic of China; 3 Department of Rheumatology and Immunology, The Affiliated Drum Tower Hospital of Nanjing University Medical School, Nanjing, 210000 People’s Republic of China

**Keywords:** Cell therapy, Umbilical cord mesenchymal stromal cells, Heterogeneity, Differentiation, Immunomodulation, Liver fibrosis

## Abstract

**Background:**

To investigate the heterogeneities of human umbilical cord mesenchymal stromal cells (HUCMSCs) derived from different donors and their therapeutic variations when applied to mouse liver fibrosis model.

**Methods:**

The characteristics of HUCMSCs derived from multiple donors were comprehensively analyzed including expressions of surface markers, viability, growth curve, karyotype analysis, tumorigenicity, differentiation potentials, and immune regulation capability. Then, the HUCMSCs with distinct immunomodulatory effects were applied to treat mouse liver fibrosis and their therapeutic effects were observed.

**Results:**

The HUCMSCs derived from multiple donors kept a high consistency in surface marker expressions, viability, growth curve, and tumorigenicity in nude mice but had robust heterogeneities in differentiation potentials and immune regulations. In addition, three HUCMSC lines applied to mice liver fibrosis model had different therapeutic outcomes, in line with individual immune regulation capability.

**Conclusion:**

The HUCMSCs derived from different donors have individual heterogeneity, which potentially lead to distinct therapeutic outcomes in mouse liver fibrosis, indicating we could make use of the donor-variation of MSCs to screen out guaranteed general indicators of MSCs for specific diseases in further stromal cell therapy.

**Supplementary Information:**

The online version contains supplementary material available at 10.1186/s13287-021-02430-6.

## Background

Mesenchymal stromal cells (MSCs) [1] have been widely tested for treating a variety of refectory medical indications such as type 1 diabetes mellitus, systemic lupus erythematosus, rheumatoid arthritis, and Crohn’s disease due to their multiple differentiation potentials and immunomodulation capability [2–6]. MSCs could be derived from a series of tissues including but not limited to umbilical cord, placenta, adipose tissue, bone marrow, gingiva, and dental pulp [6–13]. These MSCs derived from different tissues do not have uniform characteristics, differing in expression profiles of surface markers and biological functions after certain stimulation such as pro-inflammatory mediators [6, 14].

The heterogeneity of MSCs discussed above hampers the comparison of the therapeutic among different MSC products when applied to clinic use. To achieve a general definition of MSCs, the International Society for Cellular Therapy (ISCT) raised a minimal set of standard to specify MSCs as following: (i) MSCs present plastic adherence in standard culture conditions; (ii) MSCs should positively express surface markers of CD73, CD90, CD105, and negatively express CD14/CD11b, CD34, CD45, CD79α/CD19, and human leukocyte antigen (HLA)-DR; and (iii) MSCs maintains the differentiation potentials of adipocytes, osteoblasts, and chondroblasts in vitro [15]. But these criteria of MSCs have been debated ever since and each MSC product is deemed to be unique. In spite of tissue origin, many other elements such as culture method and further modulations also influence the heterogeneity of MSCs, leading to differential gene expression profiles, growth phenotype, and differentiation potentials [16–18].

Over the past few years, MSCs researches have achieved some inspiring results and some of which moved up to clinic period from preclinical phases, resulting in the marketing approval of a few cell-based therapy products (CTPs) by different national regulatory authorities [19]. According to China’s new regulatory policies, CTPs will be classified as biological drug and be regulated according to the principles of drug review and monitoring [20]. CTPs are considered to be the most complicated healing drugs in the history of human medical care due to their intricate biological features. MSCs, if considered a CTP in therapeutic use, have a huge challenge to achieve stable and uniform biological characteristics for ensuring safety and effectiveness in patients received MSC treatment.

Our hospital is approved to be qualified for implementing MSC clinic trials by government agencies [20]. We established a good manufacturing practice (GMP) grade cell facility to produce clinic-grade human umbilical cord-derived MSCs (HUCMSCs) for treating premature ovarian failure (POF) and recurrent uterine adhesion [19, 21]. In our practice of MSCs-based therapy, we set up a quality evaluation system to guarantee the security of therapeutic HUCMSCs as a CTP in clinic [22]. This quality evaluation system assayed various biological features of MSCs including but not limited to cell viability, proliferation, apoptosis, growth curve, differentiation potentials, karyotype analysis, expression of surface markers, tumorigenicity, and immunoregulation ability. In this study, based on the quality evaluation of HUCMSCs derived from each donor, we compared the heterogeneities of HUCMSCs derived from different donors and tested their therapeutic effects in mouse liver fibrosis model. Results showed HUCMSCs derived from multiple donors had remarkable individual heterogeneities in differentiation potentials and immune regulations. We proposed that MSCs with individual heterogeneity could display functional variations when applied to certain disease treatment, by which we could make use of the donor-variation of MSCs to screen out guaranteed general indicators of MSCs for specific diseases in further MSCs therapy. Thus, based on the individual immunoregulatory heterogeneity, we screened out three HUCMSC strains with different immune phenotype and applied them to mouse tetrachloromethane (CCl_4_)-induced live fibrosis treatment to examine their therapeutic efficacy. As we expected, though all three test strains of MSCs displayed effective outcomes in treating mouse liver fibrosis. MSCs owing distinct immune phenotypes had distinct therapeutic efficacy. The MSC strain with high regulatory T cells (Tregs) promotion phenotype had the best therapeutic outcomes in treating the mouse liver fibrosis by altering the endogenous T subset differentiation. Thus, we could take the advantage of individual heterogeneity to screen out seeding cells with the best criteria for specific disease.

## Materials and methods

This research was supported by the Research Ethics Board of Nanjing Drum Tower Hospital. Written consent was obtained from the puerperia who are willing to donate the umbilical cords for isolating MSCs after childbirth.

### Donor screening criteria

Cell-based therapies have the probability to spread infectious diseases. The delivery type, age and health condition of the donor can as well influence the quality and function of MSCs. Therefore, a strict donor screening is required to be carried out before sampling, including physical examination, explicit medical history, and infectious disease detection. For the sake of excluding the window phase of viral infections, 3 months after the sample donation, we will have another serological test of infectious diseases for the donor three months after umbilical cord donation [23]. The general features of umbilical cord donors are listed in supplementary table 1.

### HUCMSCs culture

MSCs are manufactured in clean environments in accordance with requirements of current GMP (cGMP) [19]. The critical raw materials and reagents applied in MSCs culturing containing fetal bovine serum (FBS, Gibco, USA), tryple, and culture medium. First of all, it is necessary to guarantee that the materials and reagents applied in cell therapy are bought from capable manufacturers, which must ensure their GMP requirements, and the credentials should be gained as well. As demanded in the laws and regulations, GMP-compliant FBS can be used for preparing therapeutic grade stromal cells, but the serum must be free of bovine spongiform encephalopathy/transmissible spongiform encephalopathy (BSE/TSE). Tryple must be certified to be free from animal viruses and porcine mycoplasmas.

The primary HUCMSCs were isolated putting to use tissue explant method [23]. The detailed experimental reagents and methods could be referred to our previous research [22]. The cells of fourth generation were harvested for further studies. To avoid the differences caused by culture method, HUCMSCs from each donor were cultured by two well-trained cell culture operators in strict accordance with standard procedures.

### Cell counts and viability

The number of cells was measured by automatic cell counter (Nexcelom, cellometer Mini, USA), and trypan blue exclusion method was used for cell viability detection. Moreover, the fourth passage cells were harvested for CCK8 and cell cycle assays as a complementary experiment to describe the viability of cells. The Cell Counting Kit (Beyotime, China) was carried out according to the manufacturer’s instruction and then the growth curve was drawn. The BD Cycletest Plus DNA Reagent Kit (BD, USA) was used to determine cell cycle.

### Surface marker expressions

The final identification of cells is the first problem that requires to be settled in cell therapy products. The settings of the cell recognition criteria contribute to the data exchange among research workers and make a distinction between blended cell population. According to guidelines from Mesenchymal and Tissue Stem Cell Committee of the ISCT, MSCs have three minimal definition criteria including adhesion to plastic, expressions of specific surface markers (CD105, CD73, CD90, positive cells ≥ 95%; CD45, CD34, CD14 or CD11b, CD79a or CD19, and HLA-DR negative cells ≤ 2%), and multi-lineage differentiation potentials of adipogenesis, osteogenesis, and chondrogenesis [15]. The detailed experimental reagents and methods could be referred to our previous research [22].

### Multi-lineage differentiation assays

In regard to multi-lineage differentiation, MSCs at the fourth passage were harvested and were replated in 24-well tissue culture plate at a density of 1 × 10^4^ cells/well. The detailed experimental reagents and methods could be referred to our previous research [22]. Briefly, HUCMSCs were cultured in adipogenic, osteogenic, or chondrogenic medium (Gibco, USA) to induce adipogenesis, osteogenesis, or chondrogenesis for 21 days and stained with Oil red (Sigma-Aldrich, USA), Alizarin Red S (Sigma-Aldrich, USA), or Alcian Blue (Sigma-Aldrich, USA) to assess the adipogenic, osteogenic, or chondrogenic differentiations, respectively. The detailed experimental procedures described in the supplemental file.

### Safety evaluation of tumorigenicity and karyotype

Researches have indicated that the possibility of chromosomal abnormality of MSCs was 4% during the in vitro culturing and the tumorigenicity of stromal cells is as well a potential safety hazard in clinic application [22]. Tumorigenicity analysis was performed within severe combined immunodeficient (SCID) mice to determine that the MSCs had no tumorigenicity risk and Giemsa banding technique was adopted for karyotype analysis to verify the genetic stability of MSCs. The detailed experimental reagents and methods could be referred to our previous research [22]. Chromosomal abnormalities include the number abnormalities and morphological distortion. In terms of tumorigenicity, 18 male SCID mice were randomly assigned into three groups, received subcutaneous injection of HUCMSCs, human embryonic stem cells (HESCs) as positive control, or PBS as negative control, respectively. The tumor formation was recorded once a week for 4 months. The mice were euthanized and the main organs were sectioned for hematoxylin-eosin (H&E) staining.

### Immunomodulation assay

Increasingly evidence indicated that the immunomodulatory function of MSCs is the basis for the treatment of systemic lupus erythematous (SLE), osteoarthritis, and other diseases. It is recommended as a potency as well a release standard for advanced period clinical trials by the ISCT [24]. The detailed experimental reagents and methods could be referred to our previous research [22]. Briefly, the immunomodulatory effects of HUCMSCs on Th1 (CD3^+^ CD8^-^ IFN-γ^+^), Th17 (CD3^+^ CD8^–^ IL17A^+^), and Tregs (CD4^+^ CD25^+^ Foxp3^+^) were detected by co-culturing HUCMSCs with human peripheral blood mononuclear cells (PBMCs) in our evaluation system. Flow cytometry (BD facsariatm, USA) was used to analyze the cells, and the data was analyzed by FACS software. The detailed experimental procedures described in the supplemental file. The inhibition of Th1 or Th17 proliferation was calculated as [[1 − (The percentage of Th1 (or Th17) in MSC group)/(The percentage of Th1 (or Th17) in PBMC group] × 100%]. The calculation method of Tregs promoting proliferation was as follows: [(The percentage of Tregs in MSC group − The percentage of Tregs in IL2 stimulation group)/The percentage of Tregs in IL2 stimulation group].

### Mouse liver fibrosis model and HUCMSC treatment via open-flow microperfusion (OFM)

All the animal experiments were performed in accordance with the guidelines and regulations from the Institutional Animal Care and Use Committee of *Nanjing University*. Animal care was provided in compliance with the National Institutes of Health guide for the care and use of Laboratory animals (NIH publications No. 8023, revised 1978). Mouse liver fibrosis was induced by CCl_4_. CCl_4_ was dissolved in corn oil (V/V, 25%), which was injected into the peritoneum of 10-week mice with a dose of 1 g/kg CC1_4_. The CCl_4_ administration was performed twice a week and lasted for 8 weeks. Mice in normal group were injected same volume of corn oil into peritoneum. Fifty mice were randomly assigned into five groups, which included normal group, control group (only CC1_4_), UC5 group (CC1_4_ with UC5 MSC liver orthotopic injection), UC11 group (CC1_4_ with UC11 MSC liver orthotopic injection), and UC12 group (CC1_4_ with UC12 MSC liver orthotopic injection). The abdominal cavity was opened to confirm the success of liver fibrosis and the mice were used for subsequent experimental operations. HUCMSCs were re-suspended into PBS (2 × 10^6^/mL) and were administrated by liver orthotopic injection via OFM as follows: Mice are anesthetized, fixed on their backs, and shaved. The abdominal cavity of the mice is cut to expose the liver after epidermal disinfection. The OFM guide cannula is implanted in the left lobe of the liver, and the OFM probe is inserted into the guide cannula. Then, the guide cannula is removed and the porous section of the OFM probe is left in the liver. The OFM probe is connected to a microdialysis pump and a syringe. Fifty microliters cell suspension is injected into the liver with a flow rate of 2 μL/min. After the injection, the mouse abdominal muscle layer and skin layer are sutured separately. Three weeks later, mice are anesthetized and the abdominal cavity is open to observe efficacy.

In this study, the level of hepatic fibrosis was researched by Masson staining (BASO, China), alanine aminotransferase (ALT) (blood samples which taken from mice’ eyeballs were sent to the laboratory for testing) and immunohistochemical staining of α-SMA/Col I: slices were put at 65~80 °C for 2 h; set into xylene I, xylene II, and xylene III for 3 min in turn; and rinsed under tap water for 30 s to 1 min after immersing in anhydrous ethanol, 95% alcohol, 80% alcohol, and 75% alcohol in turn for 2 min. Incubated with 3% hydrogen peroxide at room temperature for 15 min to remove endogenous peroxidase, the antigen was repaired by pressure cooker method (1:100 dilution of antigen repair solution into ddH_2_O). Then, the slices were sealed with 5% BSA or goat serum 60 min at room temperature. After adding appropriately diluted primary antibody at 4 °C overnight, the slices rinsed with PBST and 50~100 μL secondary antibody was added to the tissue and incubated at room temperature for 30~60 min. The color was developed by DAB, the hematlignin redyeing 1~5 min, and ammonia solution returned to blue for 5~10 s. Seventy-five percent alcohol, 80% alcohol, 95% alcohol, anhydrous ethanol I, and anhydrous ethanol II were used for 3 min to dehydrate and xylene I and xylene II were used for 5 min to permeabilize the tissues. Slices were sealed by neutral resin after drying the tissues. The staining results were observed under a microscope.

### T cell subpopulation differentiation detection of splenocytes in liver fibrosis model

Spleens were removed aseptically from experimental mice, and we isolated splenocytes after filtering through 70-μm cell strainer and suspended in Roswell Park Memorial Institute (RPMI) 1640 (Gibco, USA) complete medium supplemented with 10% FBS, 100 U/ml penicillin, and 100 mg/ml streptomycin. Then, the spleen lymphocytes were collected by discontinuous 40/70% percoll gradient centrifugation and suspended again and adjusted to the proper cell number. IFN-γ, IL-17A, and Tregs were tested by flow cytometry (FCM). The detailed experimental procedures are described in the supplemental file.

### Statistical analysis

Data were showed by mean value ± standard deviation (SD) from three or more tests. The statistical analysis of data was executed using GraphPad prism 6 software (GraphPad Software, USA). The quantitative data were compared by one-way ANOVA (S-NK). *P*-value < 0.05 was deemed statistically meaning.

## Results

### HUCMSCs stably expressing surface markers

**Fig. 1:**
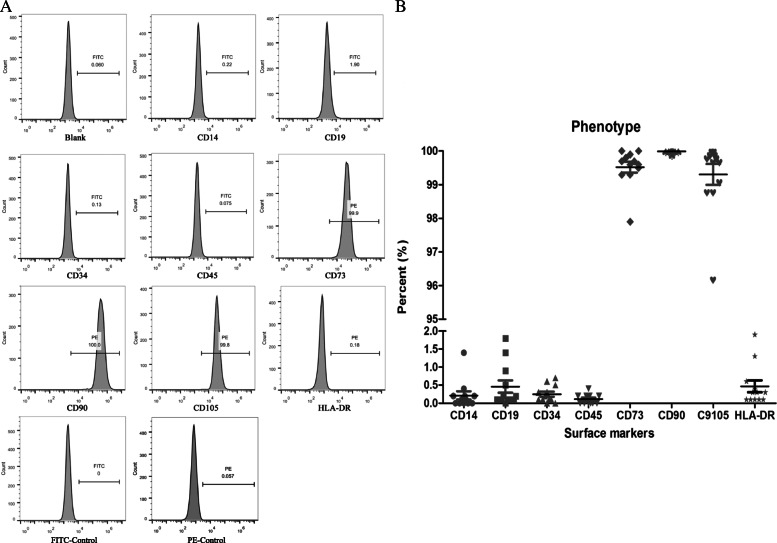
Surface markers of HUCMSCs. **A** Flow cytometry was used to detect the surface markers in HUCMSCs, CD14, CD19, CD34, CD45, and HLA-DR were negative, while CD73, CD90, and CD105 were positive. Representative images were shown in **A**. **B** Statistical analysis of all the mentioned markers of 12 HUCMSC strains

In present study, we isolated HUCMSCs from 12 donors and analyzed their individual heterogeneity and therapeutic effects in liver fibrosis model. First, we examine the surface markers of HUCMSCs by FCM and the results showed that HUCMSCs from 12 donors stably expressed positive surface markers of CD105, CD90, and CD73 (over 95% percentage) and negative surface markers of CD14, CD34, CD45, CD19, and HLA-DR (less than 2% percentage) (Fig. 1). The surface markers of 12 HUCMSC strains are listed in supplementary table 2.

### Viability and growth

**Fig. 2:**
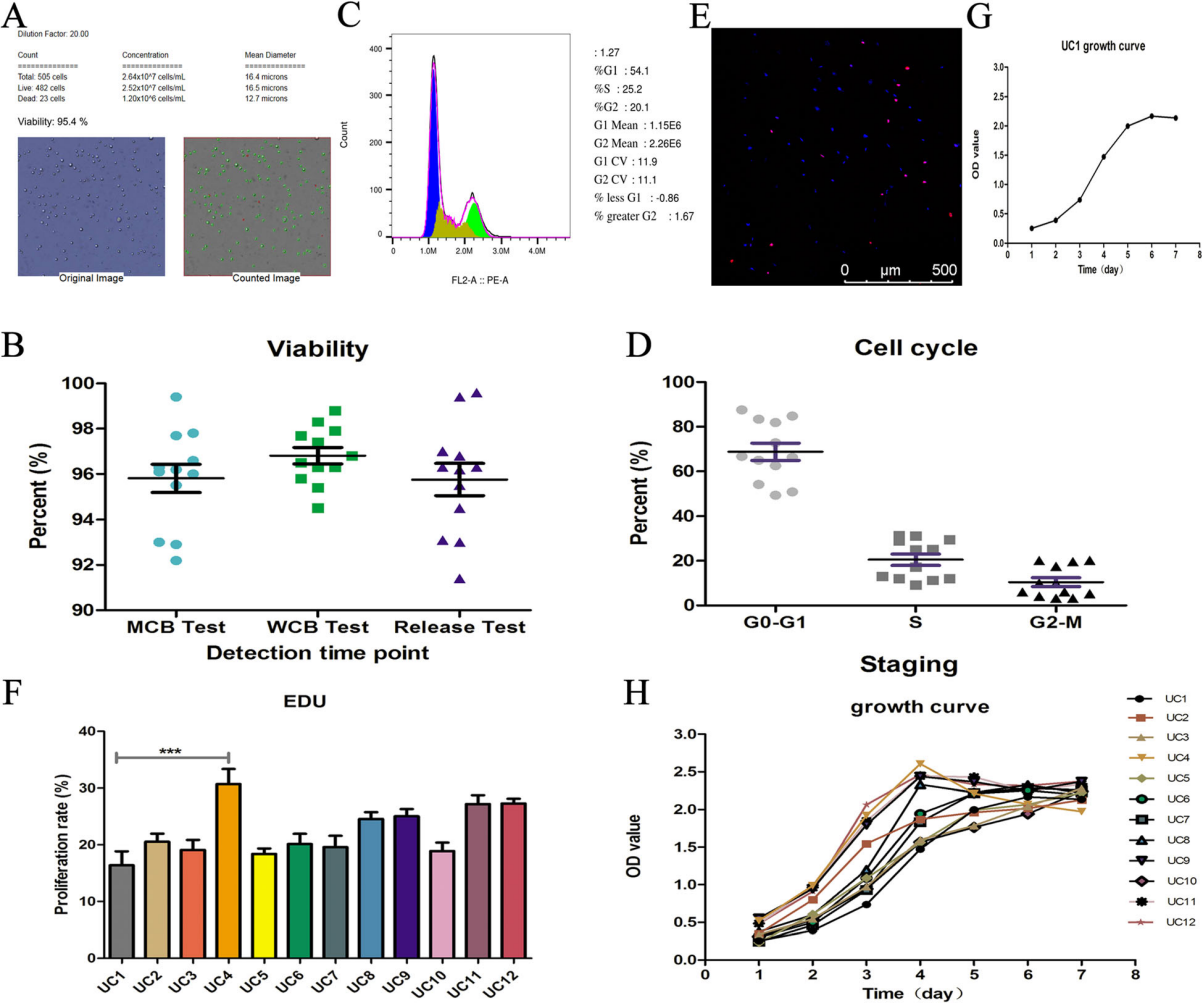
The viability and growth of 12 HUCMSCs. **A** The trypan blue exclusion data for viability. **B** The viability at different stages (Master cell bank, Working cell bank, and releasing) with a similar viability. **C**, **D** FCM analysis data for 12 HUCMSCs’ cell cycles. **E** The red nuclei cells were identified as proliferating cells in EDU immunostaining for proliferation rate. **F** There was not much difference overall among 12 HUCMSC strains. **G**, **H** HUCMCs had an “S” growth curve (***p < 0.001)

The viability and growth are two important characteristics of MSCs. The trypan blue exclusion method was performed to assay the viability at different stages (master cell bank, working cell bank, and releasing) and results showed a similar viability (over 90%) among 12 HUCMSC strains (Fig. 2A, B) which was much better than the national recommended standards for stem cell viability (> 85%) [25]. We carried out cell cycle assays (Fig. 2C, D), EDU immunostaining (Fig. 2E, F), and CCK8 (Fig. 2G, H) to examine the growth of HUCMSCs. The viability and cell cycle testing results of 12 HUCMSC strains are listed in supplementary table 3. Cell cycle analysis showed that the G1, S, and G2 phase in 12 HUCMSC strains accounted for 68.75 ± 13.46%, 20.52 ±8.77%, and 10.45 ± 6.95%, respectively (Fig. 2D). Further EDU staining revealed that there was not much difference overall among 12 HUCMSC strains, only UC4 HUCMSCs had a markedly higher proliferation rate than UC1 HUCMSCs (*p* < 0.001) (Fig. 2F). CCK8 assays showed there was certain similarity in growth curve and no significant difference from day 1 to day 7 among 12 HUCMSC strains (Fig. 2G). As is known to all, cell increases its size during the G0–G1 stage, synthesizes DNA in the S phase and synthesizes proteins to prepare for cell division during G2-M. Though the cell cycle analysis (G0/G1 vs. S vs. G2/M phases) in Table S2 shows some substantial variation between different donors, the viability and EDU were mainly the same. Judging from above results, the detection of cell cycle may not be the necessary experiment of MSCs quality evaluation.

### HUCMSCs having vast individual heterogeneity of osteogenic differentiation

**Fig. 3:**
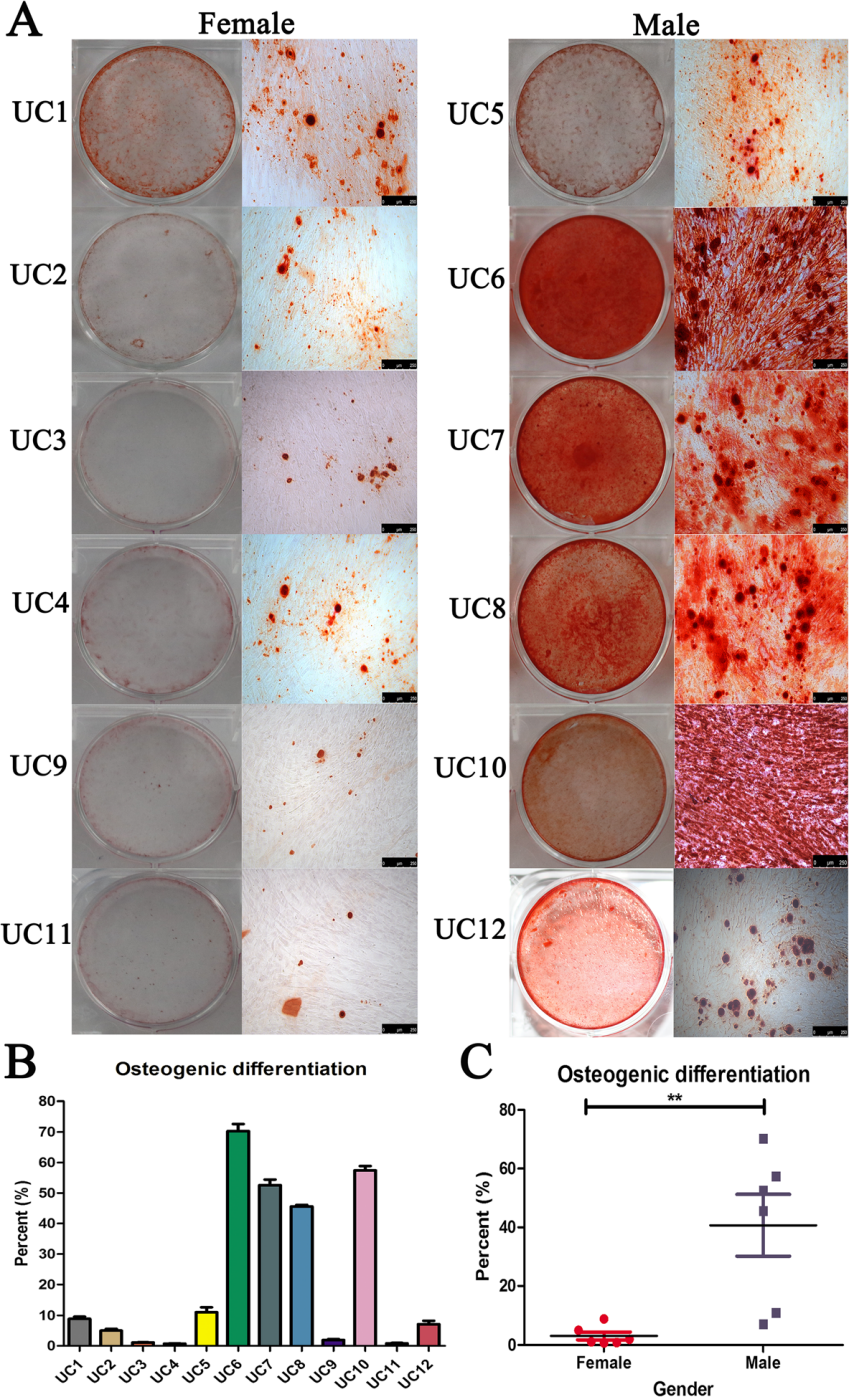
Alizarin red-S staining showed HUCMSCs were induced into osteogenic. There was a vast individual heterogeneity among 12 HUCMSC strains in osteogenic differentiation assay. HUCMSCs from male infants had significantly higher potential of osteogenesis in vitro than HUCMSCs from female infants (**p < 0.01)

**Fig. 4:**
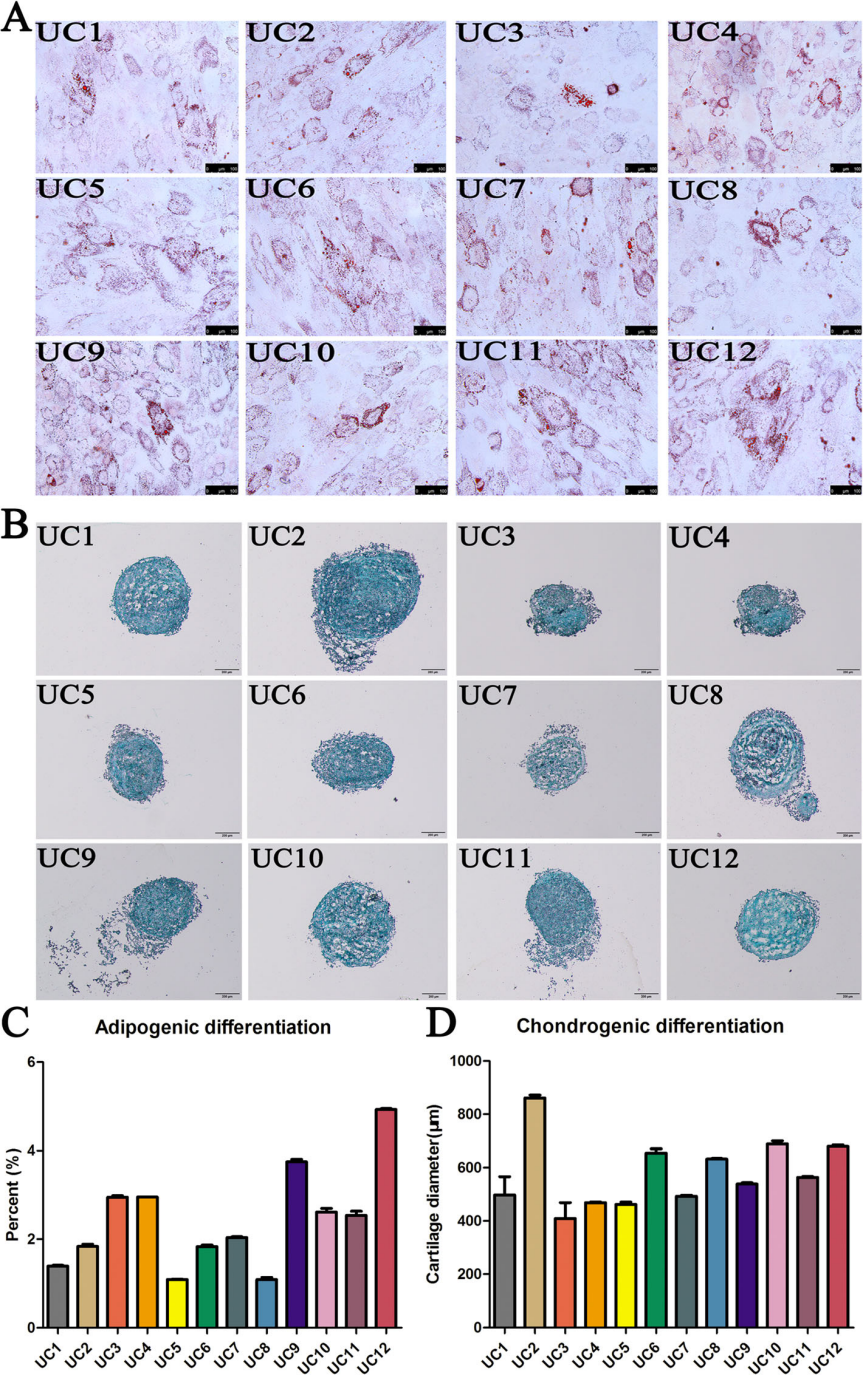
All the 12 strains of HUCMSCs were induced into adipocytes and chondrocytes detected by oil red O staining and alcian blue staining, respectively. There was no distinct individual heterogeneity in adipogenesis and chondrogenesis in vitro

It is well known that MSCs have differentiation potentials including osteocytes, adipocytes, and chondrocytes as their crucial characteristics. The osteogenic differentiation assay showed there was a vast individual heterogeneity among 12 HUCMSC strains, varying from 1 to 70% osteogenesis (Fig. 3A, B). We wondered whether gender could affect the osteogenic differentiation of HUCMSCs. The quantified data displayed that HUCMSCs from male infants had significantly higher potential of osteogenesis in vitro (about 10 folds) than HUCMSCs from female infants (*p* < 0.01), although there was also obvious heterogeneity among the 6 HUCMSC strains from male infants (Fig. 3C). In robust contrast, there was no distinct individual heterogeneity in adipogenesis and chondrogenesis in vitro (Fig. 4).

### Consistency in tumorigenicity and karyotype analysis among 12 HUCMSC strains

The tumorigenesis risk is a major concern for MSCs application in clinic. The SCID mice were subcutaneously injected HUCMSCs to monitor the tumor formation during a 4-month observation period. HESC injection was positive control and tumor was formed within about 1 month after transplantation. Among the transplantation of 12 HUCMSC strains, there was no tumorigenicity and no observation of tumor cells infiltration by H&E staining at injection sites and main organs such as heart, liver, spleen, lung, muscle, and kidney (Supplementary Fig. 1A, 1B and Supplementary table 4). In karyotype analysis, all test HUCMSCs have normal karyotype of 46 chromosomes (XX/XY) and stable genetic stability including normal morphology, number, length, size, centromere position in karyotypes, without any abnormality in deletion, reduplication, inversion, translocation, insertion, and Ring-chromosome (Supplementary Fig. 1C and Supplementary Table 5). These results displayed that there was consistency in tumorigenicity and karyotype analysis among 12 HUCMSC strains.

### Remarkable individual heterogeneity in immunomodulation effects of HUCMSCs

**Fig. 5:**
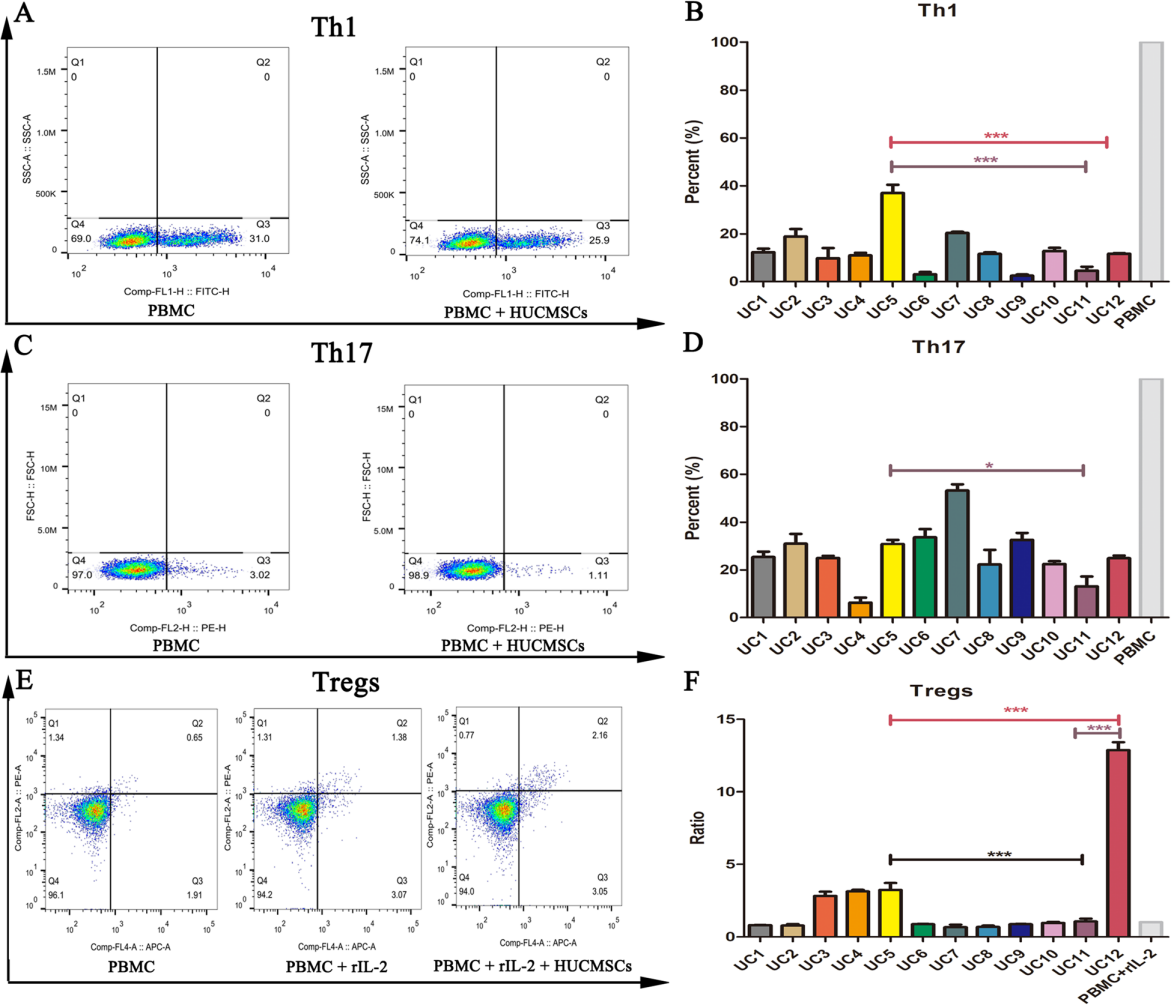
HUCMSCs inhibited the differentiations of CD4^+^ T cells into Th1 and Th17 subsets, promoted the maturation of Tregs subpopulation in PBMCs induced by IL-2. There was remarkable individual heterogeneity in immune regulation among 12 test HUCMSCs, and the same cell strain also had a different capability in every aspect of immune regulation (*p < 0.05, ***p < 0.001)

MSCs were capable to secrete immune mediators or directly interact with immune cells in recipients so as to play a therapeutic role in various immune diseases. We detected T cell subpopulation of PBMCs after being co-cultured with each HUCMSC strain to estimate the immunoregulatory effects in vitro. HUCMSC strains significantly inhibited the activation and differentiations of CD4^+^ T cells into Th1 and Th17 subpopulations and significantly promoted the maturation of Tregs subpopulation in PBMCs induced by IL-2 (Fig. 5 and Supplementary table 6). We noted that there was remarkable individual heterogeneity in immune regulation among all test HUCMSCs, and the same cell strain also had a different capability in every aspect of immune regulation. For example, UC12 HUCMSC strain had the strongest capability to promote Treg subpopulation differentiation of PBMCs among all test HUCMSC strains, but common suppression potential of Th1 and Th17 subpopulation differentiation. UC11 HUCMSCs only had very limited capability to promote Treg subpopulation differentiation and suppress Th1 and Th17 subpopulation differentiation. UC5 HUCMSC strain had the strongest capability to inhibit Th1 subpopulations differentiation of PBMCs. We assumed the remarkable individual heterogeneity of HUCMSCs in immune regulation could lead to differential therapeutic effects in curing diseases. Thus, we applied UC5, UC11, and UC12 HUCMSC strains to mouse liver fibrosis treatment to examine their therapeutic efficacy next.

### Individual heterogeneity of HUCMSCs affecting T cell subpopulation differentiation of splenocytes in liver fibrosis model

**Fig. 6:**
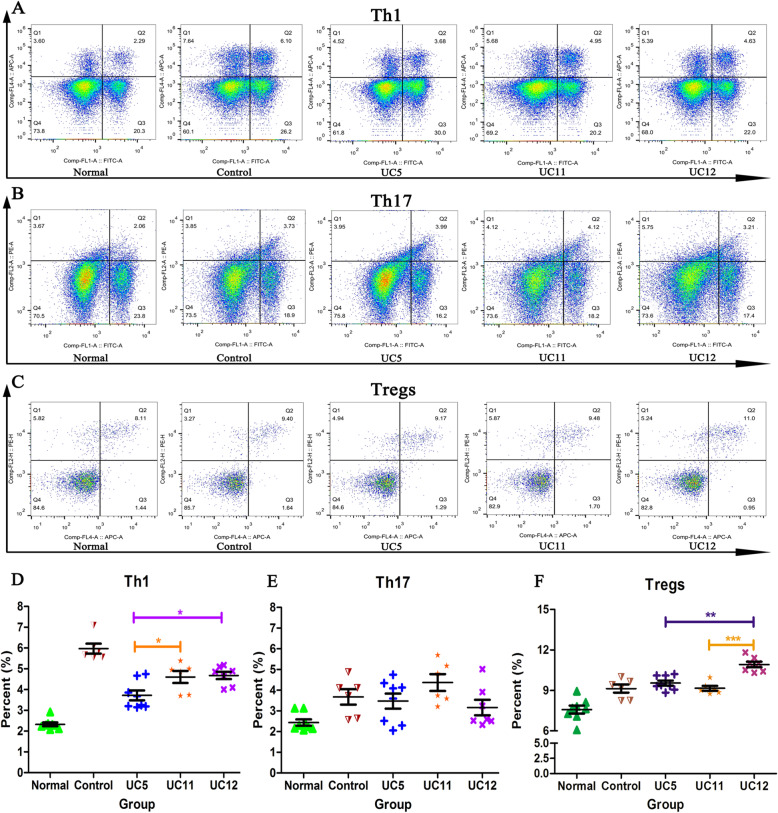
HUCMSC treatment liver fibrosis mice decreased the proportion of CD4^+^ IFN-γ^+^ T-cell (Th1) subset, compared with liver fibrosis group. UC5 HUCMSC treatment caused a lower CD4^+^ Th1 subset differentiation of splenocytes, compared with UC11 and UC12 HUCMSCs. UC12 HUCMSC treatment significantly promoted the Tregs differentiation of spenocytes, compared with UC5 and UC11 HUCMSC treatments (*p < 0.05, **p < 0.01, ***p < 0.001)

The mice were treated by CCl_4_ to induce liver fibrosis. We isolated splenocytes from control and liver fibrosis (LF) model mice to induce the differentiation of CD4^+^ T cell subsets by Cell Stimulation Cocktail ex vivo. The results showed that liver fibrosis caused a significant increase in the proportion of CD4^+^ IFN-γ^+^ T-cells (Th1), CD4^+^ IL-17A^+^ T-cells (Th17), and CD4^+^ CD25^+^ Foxp3^+^ Tregs, compared with the control group (*p* < 0.05) (Fig. 6). All three HUCMSC strains treatment decreased the proportion of CD4^+^ Th1 subset, compared with LF group (*p* < 0.05). UC5 HUCMSC treatment caused a lower CD4^+^ Th1 subset differentiation of splenocytes, compared with UC11 and UC12 HUCMSCs (*p* < 0.05). In addition, UC12 HUCMSC treatment significantly promoted the Tregs differentiation of splenocytes, compared with UC5 and UC11 HUCMSC treatments (*p* < 0.05). These results revealed that the individual heterogeneity of HUCMSCs may be capable to alter the immune responses in liver fibrosis model, which probably contributed to different therapeutic efficacy in liver fibrosis treatment.

### Individual heterogeneity resulting in differential therapeutic effects on mouse liver fibrosis

**Fig. 7:**
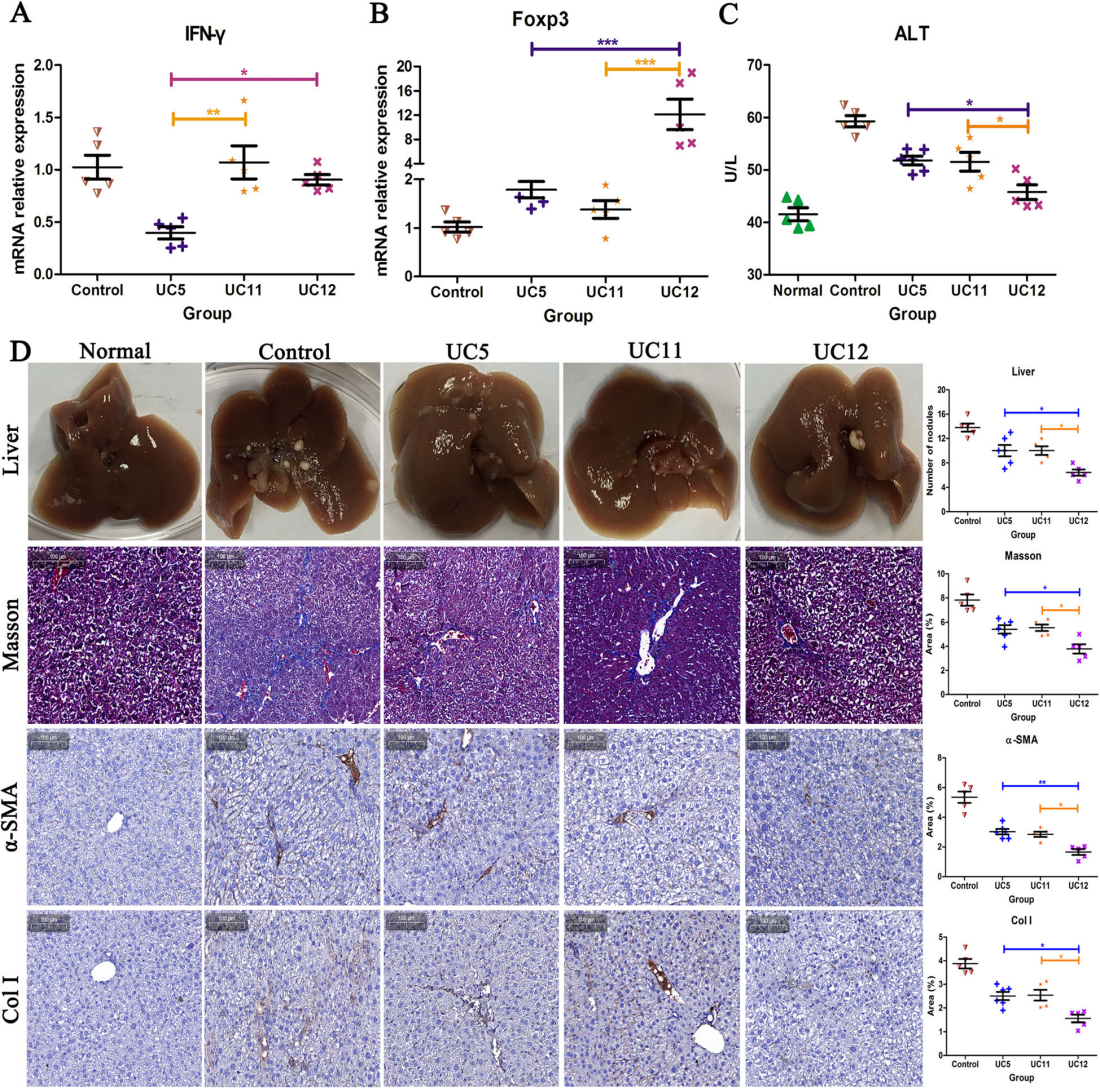
UC5 markedly decreased the IFN-γ mRNA level and UC12 significantly improved the mRNA level of Foxp3 in splenocytes, compared with LF group. UC12 HUCMSCs better increased the ALT level than other UC5 and UC11 HUCMSCs. Three HUCMSC strains markedly alleviated the amount and size of fibrosis nodules of liver surfaces, as well as the positive staining of Masson, α-SMA, and Col I in liver sections and UC12 HUCMSCs had the best therapeutic potential in liver fibrosis (*p < 0.05, **p < 0.01, ***p < 0.001)

UC5 HUCMSCs, not UC11 and UC12 HUCMSCs, markedly decreased the interferon-gamma (IFN-γ) mRNA level in splenocytes, compared with LF group (*p* < 0.05). The elevated ALT level in serum was a general marker of liver damage. Serum ALT level was elevated by CCl_4_-induced live fibrosis in mice but was markedly alleviated by all three HUCMSC strains. Further, UC12 HUCMSCs better increased the ALT level than other UC5 and UC11 HUCMSCs (*p* < 0.05). UC12 HUCMSCs, not UC5 and UC11 HUCMSCs, significantly improved about 8-fold mRNA levels of Foxp3 in splenocytes, compared with LF group (*p* < 0.001). Liver fibrosis could cause a heavy deposit of collagens in liver tissue, which lead to heavy Masson, alpha-smooth muscle actin (α-SMA), and Collagen I staining in liver tissue section. In our study, we could observe obvious nodules of liver fibrosis on the liver surface in CCl_4_ treated mice and heavily positive staining of Masson, α-SMA, and Collagen I in liver sections (Fig. 7). All three HUCMSC strains markedly alleviated the amount and size of fibrosis nodules of liver surfaces, as well as the positive staining of Masson, α-SMA, and Collagen I in liver sections. Among three HUCMSCs, UC12 HUCMSCs had the best therapeutic potential in liver fibrosis.

## Discussion

If MSCs are considered one type of drug, they beyond doubt are the most complex medicine in human medicine history due to their various biological characteristics. It is characterized by a population with significant secretion, immune regulation, and homing [1]. MSCs Committee of ISCT defines pluripotent MSCs as the minimum standard for plastic adherence, with or without specific cell surface markers, and can differentiate into multiple mesenchymal tissue lines in vitro [15]. To a large extent, these criteria are still the most basic criteria for the definition of in vitro amplification of MSCs. The main mechanism of supporting tissue regeneration and immune regulation is cell contact-dependent or -independent mechanism, mainly secreting nutritional and immunomodulatory factors [26]. The transplanted cells have only very limited long-term implantation, and there is no ectopic tissue formation [27]. Cell-based therapy products need stable and uniform biological features to achieve consistent therapeutic effects in patients received treatment. The safety of MSC products has always been the most important criterion in clinical research. However, each MSC product owns unique characteristics and biological activities, because of different donors (gender and age), origin tissue, isolation and culture method, passage number, and further modulations [16, 20]. So the standards for cell selection cannot be unified, and the treatment effects are also uneven. Therefore, we published our previous work titled “The quality evaluation system establishment of mesenchymal stromal cells for cell-based therapy products”. However, with the deepening of our research and the increase of the sample size of cell bank, we found that there is distinct individual heterogeneity among MSCs derived from same tissue origins of different donors adopted with a unified standard of measurement, because each MSC product owns unique characteristics and biological activities due to their different donors (gender and age), origin tissue, isolation and culture method, passage number, and further modulations [16, 20]. MSCs from different tissues or individuals show different phenotypes, functions, and secretory behaviors. Therefore, clinical safety needs not only to define the individual development of tissue source, but also to select the seeding cells among the cells derived from the same tissue with the best characteristics for specific disease.

In this study, we tested a series of safety-related indicators to ensure the safety characteristics of cells after transplantation into human body. The cell viability of both seed bank and working bank was more than 90%, which is higher than the 85% stipulated by ISCT [25]. EDU detection showed that the proliferation rate of our cells was generally no more than 30% in general. Compared with the 70% proliferation rate of tumor cells, none of the 12 HUCMSC lines has excessive proliferation [22]. In cell cycle detection, the S phase ratio of tumor cells is generally about 60%, while the S phase ratio of our HUCMSC is generally about 30%, which is safe compared with the over proliferation of tumor cells [22]. In addition, we tested the karyotype and tumorigenicity, and the results showed that there was no abnormality. Taking into account the differences in phenotype and function characteristics of MSC products from different tissue sources, we tested the abilities of three lineage differentiation and immune regulation of HUCMSC. Cells with strong selection ability can be selected as seed cells for the treatment of different diseases, which will enhance the effectiveness of cell therapy in vivo. All of these have established a reading for the safety screening of MSC products, and the indicators we tested for safety control are relatively comprehensive and necessary. If there is no systematic detection and comparison to screen out the optimal seed cells, it will bring risks to patients in the process of treatment. As reported in the previously literatures [28–30], MSC variation in expression of highly procoagulant tissue factor TF/CD142 expression, which is directly correlated with adverse clinical side effects and thrombotic complications/embolism to the infused cells, in particular when infused intravascularly. Nonetheless, the clinical need to improve the safety characteristics is still a top priority when giving patients in vivo treatment based on the properties of MSCs. A comprehensive understanding of product attributes, patient background, and the best mode of drug administration is essential for the safe and effective use of MSC products [26, 29, 31]. Preferential use of optimized delivery methods (e.g., careful consideration of intravascular (IV), intramuscular (IM), or endotracheal (IT) delivery methods according to product characteristics) can greatly reduce the risk of complications in patients and enable MSCs to realize their full potential.

In this study, we compared the characteristics of HUCMSCs from 12 donors using our once established systemic quality evaluation for MSCs as a cell-based therapy product [22] and found distinct individual heterogeneity among donors. Based on individual heterogeneity, we screened HUCMSCs with high Treg promotion to treat mouse liver fibrosis and obtained therapeutic benefit by altering the endogenous T subset differentiation.

In our study, all 12 strains of HUCMSCs met the standard criteria of MSCs according to ISCT proposal in 2006. All MSCs present plastic adherence in our GMP-complaint culture conditions and stably expressed positive surface markers (CD105, CD90, and CD73), and negative surface markers (CD14, CD45, CD19, CD34, and HLA-DR). Meanwhile, all strains of MSCs maintained the differentiation potentials of adipocytes, osteoblasts, and chondroblasts in vitro. In addition, all 12 strains of MSCs collectively shared similar characteristics in viability, cell cycle, growth, and proliferation.

However, we noted that there was evident individual heterogeneity among 12 strains in several vital biological properties. One hallmark of the MSCs is their multipotency to differentiate into multiple lineages. We found that there was a vast individual heterogeneity among 12 HUCMSC strains, varying from 1 to 70% osteogenesis. Moreover, HUCMSCs from male infants had robust higher potential of osteogenesis in vitro (about 10 folds) than HUCMSCs from female infants, indicating the gender remarkedly affects the osteogenesis capability of MSCs. In robust contrast, there was no distinct individual heterogeneity in adipogenesis and chondrogenesis.

To our acknowledgement, it is the first time to report the remarkable gender differences in the osteogenic differentiation ability of MSCs. This difference is not caused by proliferation capabilities of MSCs because we tested the CCK-8 and EDU proliferation abilities of 12 HUCMSC strains and did not observe statistical difference in vitro expansion between male and female. The process of osteogenic differentiation and bone regeneration of MSCs is regulated by a variety of hormones, transcription factors, and cell signaling pathways, including bone morphogenetic protein, Wnt, insulin-like growth factor, epidermal growth factor and growth hormone, and participate in the interaction of many ways [32–35]. On the one hand, we speculate that the ability of osteogenic differentiation caused by gender difference may be related to alkaline phosphatase (ALP). It has been reported that the ALP activity in male cells is significantly higher than that in female cells [36]. Before or after osteogenic stimulation, male cells have higher ALP activity than female cells, and ALP activity is widely used as a marker of osteogenesis [37]. Secondly, at the cellular level, the response of female and male cells to estrogen and androgen was different [38], which also found gender differences in the response to progesterone in the lumbar spine cells of rats [39]. The difference in steroid receptors between males and females is also an important contributor and mediator to MSCs proliferation and differentiation abilities [40]. The mechanism of sex-specific action of estrogen may be related to the difference of estrogen receptor subtypes (α and β) [41]. In fact, these nuclear receptors regulate the transcriptional activity of specific genes by recruiting a series of auxiliary activator proteins, including SRC1 (steroid receptor co-activator 1), and whose expression is reported to be gender specific [42]. Thirdly, male MSCs have more osteoprogenitor cells than female MSCs [36], which is more conducive to osteogenic differentiation, and therefore it may have higher osteogenic capacity and bone regeneration ability. We hope that future research will focus on investigating the role of cell gender in MSCs isolated from human different organizations from male and female donors, as well as from adolescent and adult donors. These future studies will expand our understanding of the applicability of MSCs for bone tissue engineering and should encourage other researchers to study and report any gender-related differences, as they may clearly have significant clinical implications. In the relevant clinical application of MSCs for bone repair, we recommend that male MSCs can be used as seed cells for local transplantation, which may have a more significant clinical therapeutic effect.

It was well known that MSCs were capable to secrete immune mediators or directly interact with immune cells to play a role of immune regulation. HUCMSC strains remarkably suppressed the activation and differentiations of CD4^+^ T cells into Th1 and Th17 subpopulations and significantly promoted the maturation of Tregs subpopulation in PBMCs induced by IL-2 in vitro. We observed that there was remarkable individual heterogeneity in immune regulation among all test HUCMSCs. Interestingly, the same cell strain also had a different capability in every aspect of immune regulation. For example, UC12 HUCMSC strain had the strongest capability to promote Treg subpopulation differentiation of PBMCs among all test HUCMSC strains, but mean suppression potential of Th1 and Th17 subpopulation differentiation. Accumulating evidences have showed that human MSCs derived from different donors have individual heterogeneity [43–45]. For example, Phinney et al. reported that there were dramatic differences in levels of bone-specific gene expressions and alkaline phosphatase enzyme activity among MSC populations derived from posterior iliac crest marrow of 17 healthy donors [46]. Summarily, the subtle intrinsic variability in MSC populations derived from different donors is a common phenomenon, apart from the inconformity of cultivation procedure and conditions.

Undoubtedly, the intrinsic individual heterogeneity in MSC populations is a major obstacle for obtaining consistent cell-based therapeutic products aiming at MSCs. We proposed that MSCs with individual heterogeneity could display functional variations when applied to certain disease treatment. Thus, we could make use of the donor-variation of MSCs to screen out guaranteed general indicators of MSCs for specific diseases in further stem cell therapy. Many of signaling molecules between human and rodent are evolutionarily highly conserved. Thus, the certain animal model including rodents generally is applied to test the safety and effectiveness of cell-based therapeutic product before clinic application. For example, clinical grade HUCMSCs play an important role in the repair of hippocampal neurons in SAMP8 mice (an accelerated aging mouse model of Alzheimer’s disease) by the secretion of core functional factor HGF [47]. The main mechanism of MSC action in supporting tissue regeneration and immunoregulation is mainly through the secretion of nutritional and immunoregulatory factors [26]. Based on the individual immunoregulatory heterogeneity, we screened UC5, UC11, and UC12 strains of MSCs with different immune phenotype and applied them to mouse CCl4-induced live fibrosis treatment to examine their therapeutic efficacy.

As we expected, though all three test strains of MSCs displayed effective outcomes in treating mouse liver fibrosis, MSCs derived from different donors, owing distinct immune phenotypes, had distinct therapeutic efficacy. Liver fibrosis could cause a heavy deposit of collagens in liver tissue, which lead to heavy Masson, α-SMA, and Collagen I staining in liver tissue section. All three HUCMSC strains markedly alleviated the amount and size of fibrosis nodules of liver surfaces, as well as the positive staining of Masson, α-SMA, and Collagen I in liver sections. Among three HUCMSCs, UC12 HUCMSCs had the best therapeutic potential in liver fibrosis. UC12 strain of MSCs had the highest potential of Treg cell differentiation and we also observed UC12 strain of MSCs could promote Treg differentiation in phenotype, proving the heterogeneity of MSCs owned the functional discrepancy in disease treatment.

MSCs have the potential of liver differentiation, immunomodulatory function, and the ability to produce nutritional factors, making them ideal drugs for the treatment of liver fibrosis [48]. A number of animal studies have shown that MSCs can safely reverse liver fibrosis and improve liver function [49–51]. In recent years, the immunomodulatory function of MSCs has gradually become the main research target in the treatment of liver fibrosis. In vitro experiments have proved the effects of HUCMSCs on different immune cell subsets while these effects are still need to be confirmed in animal models of liver fibrosis [48]. We selected three strains of HUCMSCs, with significantly different in vitro immune regulation capability. Among them, UC12 has the highest immune regulation capability of Tregs subsets in vitro, and exerts the strongest anti-fibrosis effect in the mouse liver fibrosis model induced by carbon tetrachloride. It is consistent with the anti-fibrosis effect of Tregs reported in many literatures. Claassen et al. found that there were a large number of Treg cells in the liver of hepatitis C-related liver fibrosis model, and the degree of liver fibrosis decreased with the increase of the number of Treg cells, suggesting that Tregs had an inhibitory effect on the formation of liver fibrosis [52]. Treg cells are a subgroup of CD4^+^ T cells with reverse regulatory function. It plays an irreplaceable role in the immune tolerance and over-effect regulation by inhibiting the inflammatory stimulation response, preventing the excessive effect response and maintaining the immune balance. On the one hand, we speculate that the orthotopic transplanted MSCs secrete many soluble factors (such as prostaglandin E2, IDO, and IL-10) in the liver, which can change the microenvironment, exert anti-inflammatory effect, eliminate effector cells, significantly inhibit hepatocyte apoptosis, and promote the proliferation of hepatic cell, so as to achieve the purpose of protecting liver tissue [53]. On the other hand, MSCs exert the effect of systemic immunity and changes the proportion of immune effector cells in mice. The increased Tregs cell subsets reduce the infiltration of CD8^+^ lymphocytes into the liver, decrease the level of pro-inflammatory factors such as tumor necrosis factor-α in circulation, inhibit the proliferation of activated hepatic stellate cells and collagen synthesis, and finally reduces fibrosis [54]. Hence, compared with the other two cell strains, UC12 improved the immune subsets of Tregs in mice to a greater extent, and the degree of liver fibrosis was significantly reversed.

There were some limitations in this study. We chose three MSC phenotypes (UC5, 11, 12) to test their therapeutic effect on in vivo fibrosis model. We observed MSCs with higher Treg promotion have improved properties to reduce fibrosis, but only repeated measurements of the same product. This result has only been shown for one donor and could thus be a pure finding of chance. We should further screen out more MSC strains and validate statistically relevant differences between groups of multiple donors with similar phenotypes in each group.

Our study revealed that although MSCs are derived from same tissue origin such as human umbilical cord, they own donor-related heterogeneity, which could contribute to the explanation of experimental and clinical discrepancy. It is a huge challenge for researchers to obtain consistent qualified MSCs in clinic use because of the individual heterogeneity. On other hand, we could take the advantage of individual heterogeneity to screen seeding cells with the best criteria for certain disease treatment. We could establish an optimized criterion, beyond the general standards of MSCs to provide therapeutic benefit for various diseases, according to the disease pathogenesis, mechanism, and process. For example, the MSCs derived from male infant with high potential of osteogenesis could be applied to recover the huge bone defect. MSCs with a strong immunoregulatory effect were screened out for treating immune-related diseases.

## Conclusion

Above all, we first proposed that MSCs should be established grading standards as a cell-based therapeutic product. The class standard is the general standard of MSCs as ISCT proposed, including certain surface marker expressions, plastic adherence, differentiation potentials of adipocytes, osteoblasts, and chondroblasts in vitro, as well as immune phenotypes. The class II standard should go beyond the general standard of MSCs, namely the internal standards in each manufacture. The class III standard of MSCs is established for specific disease therapy based on the individual heterogeneity screening according to the disease pathogenesis, mechanism, and process, as well the strategy of therapy.

## Supplementary Information

**Additional file 1: Supplementary Table 1.** The general features of umbilical cord donors. **Supplementary Table 2.** The surface markers of HUCMSCs. **Supplementary Table 3.** The viability and cell cycle testing results of HUCMSCs. **Supplementary Table 4.** Tumorigenicity of HUCMSCs. **Supplementary Table 5.** Chromosome karyotype analysis therapeutic efficacy next. **Supplementary Table 6.** Th1 and Th17 suppression, Treg promotion of PBMCs influenced by HUCMSCs strains

**Additional file 2.** Supplement of materials and methods.

**Additional file 3: Supplementary Figure 1.** Tumorigenicity and karyotype analysis among 12 HUCMSCs strains. A: The tumor formation was observed in HESCs injected mice, but there was no tumorigenicity and observation of tumor cells infiltration by H&E staining at injection sites skin and muscle among the transplantation of 12 HUCMSCs strains and PBS control mice. B: Main organs showed no abnormality detected by H&E staining in HUCMSCs and PBS group mice. C: In karyotype analysis, all test HUCMSCs have normal karyotype of 46 chromosomes (XX/XY) and stable genetic stability.

## Data Availability

The datasets used and/or analyzed during the current study are available from the corresponding author on reasonable request.

## Abbreviations

MSCs: Mesenchymal stromal cells
ISCT: The International Society for Cellular Therapy
HLA: Human leukocyte antigen
CTP: Cell-based therapy product
GMP: Good manufacturing practice
HUCMSCs: Human umbilical cord mesenchymal stromal cells
POF: Premature ovarian failure
CCl_4_: Tetrachloromethane
Tregs: Regulatory T cells
FBS: Fetal bovine serum
BSE: Bovine Spongiform Encephalopathy
TSE: Transmissible Spongiform Encephalopathy
SLE: Systemic lupus erythematous
PBMCs: Peripheral blood mononuclear cells
OFM: Open-flow microperfusion
ALT: Alanine aminotransferase
H&E: Hematoxylin-eosin
RPMI: Roswell Park Memorial Institute
FCM: Flow cytometry
SD: Standard deviation
LF: Liver fibrosis
PBS: Phosphate-buffered saline
SCID: Severe combined immune deficiency
HESCs: Human embryonic stem cells
ALP: Alkaline phosphatase
SRC1: Steroid receptor co-activator 1
