# Supplementary material for: Individual heterogeneity screened umbilical cord-derived mesenchymal stromal cells with high Treg promotion demonstrate improved recovery of mouse liver fibrosis
Source: Stem Cell Res Ther. 2021 Jun 22;12:359. doi: 10.1186/s13287-021-02430-6 (PMC8220795; doi:10.1186/s13287-021-02430-6)
Supplement: Supplementary file 1 — Additional file 1: Supplementary Table 1. The general features of umbilical cord donors. Supplementary Table 2. The surface markers of HUCMSCs. Supplementary Table 3. The viability and cell cycle testing results of HUCMSCs. Supplementary Table 4. Tumorigenicity of HUCMSCs. Supplementary Table 5. Chromosome karyotype analysis therapeutic efficacy next. Supplementary Table 6. Th1 and Th17 suppression, Treg promotion of PBMCs influenced by HUCMSCs strains [file 13287_2021_2430_MOESM1_ESM.docx]

Supplementary table 1. The general features of umbilical cord donors

|  | Age | Gestation | Delivery mode | Parity | Neonate gender |
| --- | --- | --- | --- | --- | --- |
| UC1 | 32 | 39+5 | Vaginal | 2 | Female |
| UC2 | 25 | 36+6 | Vaginal | 1 | Female |
| UC3 | 26 | 38+3 | Vaginal | 1 | Female |
| UC4 | 31 | 37+3 | Vaginal | 1 | Female |
| UC5 | 32 | 38+2 | Vaginal | 1 | Male |
| UC6 | 29 | 38+2 | Vaginal | 3 | Male |
| UC7 | 29 | 40 | Vaginal | 1 | Male |
| UC8 | 28 | 39+4 | Vaginal | 1 | Male |
| UC9 | 27 | 39+4 | Vaginal | 1 | Female |
| UC10 | 26 | 38+3 | Vaginal | 1 | Male |
| UC11 | 28 | 39 | Vaginal | 1 | Female |
| UC12 | 25 | 40+2 | Vaginal | 1 | Male |

Supplementary table 2. The surface markers of HUCMSCs

|  | UC1 | UC2 | UC3 | UC4 | UC5 | UC6 | UC7 | UC8 | UC9 | UC10 | UC11 | UC12 |
| --- | --- | --- | --- | --- | --- | --- | --- | --- | --- | --- | --- | --- |
| CD14 | 0.2 | 0.0 | 0.1 | 0.0 | 0.2 | 1.4 | 0.4 | 0.1 | 0.0 | 0.0 | 0.0 | 0.2 |
| CD19 | 1.8 | 0.0 | 0.1 | 0.2 | 0.1 | 0.5 | 0.2 | 0.9 | 1.4 | 0.1 | 0.1 | 0.1 |
| CD34 | 0.1 | 0.5 | 0.3 | 0.2 | 0.1 | 0.7 | 0.3 | 0.1 | 0.6 | 0.0 | 0.0 | 0.1 |
| CD45 | 0.2 | 0.0 | 0.0 | 0.2 | 0.2 | 0.4 | 0.2 | 0.0 | 0.0 | 0.0 | 0.0 | 0.2 |
| CD73 | 100.0 | 99.6 | 100.0 | 99.8 | 99.3 | 97.9 | 99.6 | 99.9 | 99.7 | 99.7 | 99.5 | 99.3 |
| CD90 | 100.0 | 99.9 | 100.0 | 100.0 | 100.0 | 100.0 | 100.0 | 100.0 | 100.0 | 100.0 | 100.0 | 100.0 |
| CD105 | 99.8 | 99.8 | 99.7 | 96.2 | 98.8 | 99.1 | 99.7 | 99.9 | 99.9 | 100.0 | 100.0 | 98.8 |
| HLA-DR | 0.1 | 1.3 | 0.1 | 0.4 | 0.1 | 1.9 | 0.3 | 0.3 | 0.6 | 0.3 | 0.1 | 0.1 |

Supplementary table 3. The viability and cell cycle testing results of HUCMSCs

|  | | UC1 | UC2 | UC3 | UC4 | UC5 | UC6 | UC7 | UC8 | UC9 | UC10 | UC11 | UC12 | **‾**X±SD |
| --- | --- | --- | --- | --- | --- | --- | --- | --- | --- | --- | --- | --- | --- | --- |
| Viability (%) | MCB | 96.30 | 95.50 | 97.80 | 93.00 | 96.10 | 97.70 | 96.00 | 92.90 | 92.20 | 99.40 | 96.60 | 96.20 | 95.81±2.15 |
|  | WCB | 95.40 | 97.90 | 96.80 | 97.70 | 97.40 | 95.80 | 96.30 | 98.30 | 96.30 | 98.80 | 94.50 | 96.50 | 96.81±1.26 |
| Cell Cycle (%) | G0/G1 | 84.78 | 62.54 | 66.28 | 72.79 | 81.90 | 83.37 | 87.54 | 64.94 | 66.69 | 50.80 | 54.10 | 49.30 | 68.75±13.46 |
|  | S | 12.04 | 31.34 | 29.56 | 17.21 | 12.09 | 11.32 | 9.23 | 25.06 | 13.10 | 31.10 | 25.20 | 29.00 | 20.52±8.77 |
|  | G2/M | 3.18 | 6.13 | 4.16 | 10.00 | 6.01 | 5.31 | 3.23 | 10.00 | 20.21 | 17.50 | 20.10 | 19.60 | 10.45±6.95 |

MCB: Master Cell Bank; WCB: Working Cell Bank.

Supplementary table 4. Tumorigenicity of HUCMSCs

|  | UC1 | UC2 | UC3 | UC4 | UC5 | UC6 | UC7 | UC8 | UC9 | UC10 | UC11 | UC12 | PBS control | HESCs control |
| --- | --- | --- | --- | --- | --- | --- | --- | --- | --- | --- | --- | --- | --- | --- |
| Skin | - | - | - | - | - | - | - | - | - | - | - | - | - | + |
| Muscle | - | - | - | - | - | - | - | - | - | - | - | - | - | + |
| Heart | - | - | - | - | - | - | - | - | - | - | - | - | - | - |
| Liver | - | - | - | - | - | - | - | - | - | - | - | - | - | - |
| Spleen | - | - | - | - | - | - | - | - | - | - | - | - | - | - |
| Lung | - | - | - | - | - | - | - | - | - | - | - | - | - | - |
| Kidney | - | - | - | - | - | - | - | - | - | - | - | - | - | - |
| Ovary | - | - | - | - | - | - | - | - | - | - | - | - | - | - |
| Uterus | - | - | - | - | - | - | - | - | - | - | - | - | - | - |
| Ovary | - | - | - | - | - | - | - | - | - | - | - | - | - | - |

Supplementary table 5. Chromosome karyotype analysis

|  | UC1 | UC2 | UC3 | UC4 | UC5 | UC6 | UC7 | UC8 | UC9 | UC10 | UC11 | UC12 |
| --- | --- | --- | --- | --- | --- | --- | --- | --- | --- | --- | --- | --- |
| Karyotype | XX | XX | XX | XX | XY | XY | XY | XY | XX | XY | XX | XY |
| Number | 46 | 46 | 46 | 46 | 46 | 46 | 46 | 46 | 46 | 46 | 46 | 46 |
| Morphology | N | N | N | N | N | N | N | N | N | N | N | N |
| Length | N | N | N | N | N | N | N | N | N | N | N | N |
| Size | N | N | N | N | N | N | N | N | N | N | N | N |
| The centromere position | N | N | N | N | N | N | N | N | N | N | N | N |
| Deletion | N | N | N | N | N | N | N | N | N | N | N | N |
| Reduplication | N | N | N | N | N | N | N | N | N | N | N | N |
| Inversion | N | N | N | N | N | N | N | N | N | N | N | N |
| Translocaion | N | N | N | N | N | N | N | N | N | N | N | N |
| Insertion | N | N | N | N | N | N | N | N | N | N | N | N |
| Ring-chromosome | N | N | N | N | N | N | N | N | N | N | N | N |

Supplementary table 6. Th1 and Th17 suppression, Treg promotion of PBMCs influenced by HUCMSCs strains

|  | UC1 | UC2 | UC3 | UC4 | UC5 | UC6 | UC7 | UC8 | UC9 | UC10 | UC11 | UC12 |
| --- | --- | --- | --- | --- | --- | --- | --- | --- | --- | --- | --- | --- |
| Th1 suppression (%) | 12.32±2.09 | 18.89±4.37 | 9.76 ±6.11 | 11.00±1.46 | 36.97±4.87 | 3.08±1.26 | 20.33±0.77 | 11.59±0.91 | 2.46±0.77 | 12.78±1.94 | 4.57±2.34 | 11.67±0.35 |
| Th17 suppression (%) | 23.50±3.26 | 30.96±5.78 | 24.93 ±1.33 | 6.26±2.96 | 30.80±2.46 | 33.63±4.83 | 53.13±3.67 | 22.21±8.74 | 32.56±4.14 | 22.45±1.61 | 13.07±5.87 | 24.89±1.56 |
| Tregs promotion (ratio) | 0.80±0.03 | 0.77±0.13 | 2.81 ±0.41 | 3.13±0.16 | 3.23±0.68 | 0.86±0.04 | 0.65±0.26 | 0.67±0.14 | 0.86±0.04 | 0.95±0.11 | 1.05±0.28 | 12.87±0.77 |
