# Supplementary material for: Individual heterogeneity screened umbilical cord-derived mesenchymal stromal cells with high Treg promotion demonstrate improved recovery of mouse liver fibrosis
Source: Stem Cell Res Ther. 2021 Jun 22;12:359. doi: 10.1186/s13287-021-02430-6 (PMC8220795; doi:10.1186/s13287-021-02430-6)
Supplement: Supplementary file 2 — Additional file 2. Supplement of materials and methods. [file 13287_2021_2430_MOESM2_ESM.docx]

**Supplement of materials and methods：**

1. **Multilineage differentiation assays**

In regard to multilineage differentiation, MSCs at the fourth passage were harvested and were replated in 24-well tissue culture plate at a density of 1 × 10^4^ cells /well. When the cells reached 50 % ~ 70 % confluency, adipogenic and osteogenic medium (Gibco, USA) was replaced to induce adipogenesis and osteogenesis, respectively. After 21 days, cells were fixed in 4 % formaldehyde for 30 min and stained with Oil red (Sigma-Aldrich, USA) and Alizarin Red S (Sigma-Aldrich, USA) for 30 min to evaluate the adipogenic and osteogenic differentiations, respectively. In addition, 2 × 10^5^ cells at the fourth passage were centrifuged for 10 min at 2, 000 rpm/min in a tube and chondrogenic medium was (Gibco, USA) added in the pellet after removal of supernatant to evaluate the chondrogenic differentiation of MSCs. After 21 days, the pellet was fixed in 4 % formaldehyde, dehydrated through serial ethanol dilutions and embedded in OCT. Blocks were cut into 5-mm thickness sections and stained with Alcian Blue (Sigma-Aldrich, USA).

1. **Immunomodulation assay**

In our evaluation system, the immunomodulatory effects of HUCMSCs on Th1, Th17, and the regulatory T cells (Tregs) were assayed by co-culturing HUCMSCs with human peripheral blood mononuclear cells (PBMCs). The method was briefly described as below. HUCMSCs were plated into 6-well plates (1 × 10^5^ / well) and were incubated for 24 hours with Roswell Park Memorial Institute (RMPI) 1640 complete medium. Human PBMCs were prepared from leucopheresis packs by centrifugation on a Ficoll Hypaque density gradient (AXIS-SHIELD, NOR). PBMCs were then plated into 6-well plates in the presence or absence of HUCMSCs (HUCMSCs / PBMCs ratio, 1:10). For Tregs population assay, rhIL-2 (5 ng/mL) was needed to add to the culture medium. After co-culturing for three days, T cells were collected and stimulated for five hours with 1 × compound stimulant of cocktail（Invitrogen，USA）. Cells were first stained with CD3-Percp and CD8-APC (BD, USA) and incubated for 15 minutes at room temperature. Then cells were fixed and permeabilized using the Cell Fixation & Permeabilization kit (FMS, China) according to the manufacturer’s instructions. After washing, we performed intracellular staining for IFN-γ-FITC and IL-17A-PE detection. For the detection of Tregs, non-adherent cells were harvested and evaluated according to the manufacturer’s instructions (eBioscience, USA). Cells were analyzed through flow cytometry (BD FACSAria^TM^, USA) and data were analyzed with FACS software.

1. **T cells subpopulation differentiation detection of splenocytes in liver fibrosis model**

Spleens were removed aseptically from experimental mice, and we isolated splenocytes after filtering through 70 μm cell strainer and suspended in RPMI 1640 complete medium supplemented with 10 % bovine fetal serum, 100 U/mL penicillin, and 100 mg/Ml streptomycin and the spleen lymphocytes were collected by discontinuous 40 / 70 % percol gradient centrifugation and suspended again and adjusted to the proper cell number. The lymphocytes were stimulated with 1 × compound stimulant of cocktail（Invitrogen，USA）for 5 h. The cells were subjected to FITC-labeled anti-mouse CD4 (BD Pharmingen), followed by APC-labeled anti-mouse IFN-γ (BD Pharmingen), and PE-labeled anti-mouse IL-17A (BD Pharmingen) for intracellular cytokine staining after using the Cell Fixation & Permeabilization kit (FMS, China) according to the manufactures’ instructions. For the detection of Tregs, cells were first stained with FITC-labeled anti-mouse CD4 and APC-labeled anti-mouse CD25 (BD Pharmingen). After surface staining, the cells were stained with PE-labeled anti-mouse Foxp3 antibodies. Cells were analyzed through flow cytometry (BD FACSAria^TM^, USA) and data were analyzed with FACS software.
