# Supplementary figures and images for: Individual heterogeneity screened umbilical cord-derived mesenchymal stromal cells with high Treg promotion demonstrate improved recovery of mouse liver fibrosis
Source: Stem Cell Res Ther. 2021 Jun 22;12:359. doi: 10.1186/s13287-021-02430-6 (PMC8220795; doi:10.1186/s13287-021-02430-6)

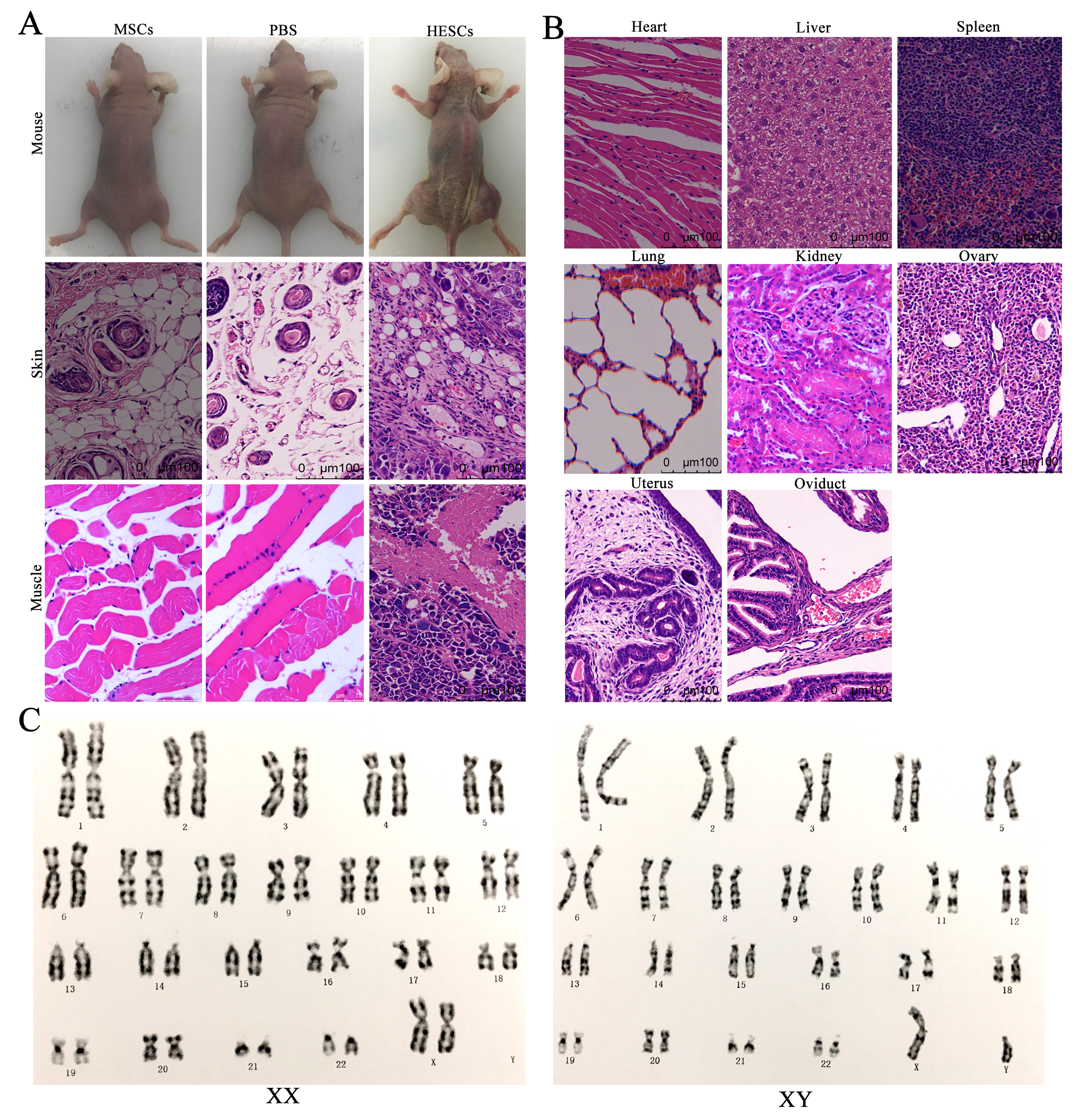

Supplement: Supplementary file 3 — Additional file 3: Supplementary Figure 1. Tumorigenicity and karyotype analysis among 12 HUCMSCs strains. A: The tumor formation was observed in HESCs injected mice, but there was no tumorigenicity and observation of tumor cells infiltration by H&E staining at injection sites skin and muscle among the transplantation of 12 HUCMSCs strains and PBS control mice. B: Main organs showed no abnormality detected by H&E staining in HUCMSCs and PBS group mice. C: In karyotype analysis, all test HUCMSCs have normal karyotype of 46 chromosomes (XX/XY) and stable genetic stability. [file 13287_2021_2430_MOESM3_ESM.tif]
